# Medication non-adherence in patients with type 2 diabetes: a cross-sectional study in Morocco

**DOI:** 10.11604/pamj.2025.52.187.48329

**Published:** 2025-12-28

**Authors:** Ibtisam Haidar, Asma Chadli, Nassim Essabah Haraj, Siham El Aziz, Malak Iggar, Imane Bounjerte, Anass Kettani, Rachid Saile, Houda Bennani

**Affiliations:** 1 Laboratory of Biology and Health, Faculty of Sciences Ben M'Sik, Hassan II University, Casablanca, Morocco,; 2 Department of Endocrinology, Diabetology, Metabolic Diseases and Nutrition, Ibn Rochd University Hospital, Casablanca, Morocco,; 3 Laboratory of Neurosciences and Mental Health, Faculty of Medicine and Pharmacy, Hassan II University, Casablanca, Morocco

**Keywords:** Type 2 diabetes, medication non-adherence, factors associated, therapeutic education, Morocco

## Abstract

**Introduction:**

medication non-adherence in patients with type 2 diabetes substantially contributes to poor glycemic control and increases the risk of complications. This study aimed to assess the prevalence of medication non-adherence and to identify its associated factors among Moroccan patients with type 2 diabetes.

**Methods:**

a cross-sectional study was conducted in the Endocrinology Department of Ibn Rochd University Hospital in Casablanca, Morocco. A total of 329 adult patients with type 2 diabetes were included. Medication adherence was evaluated using the 8-item Morisky Medication Adherence Scale (MMAS-8). Bivariate logistic regression was performed to examine associations between non-adherence and socio-demographic, clinical, treatment, and behavioral-related factors.

**Results:**

among the participants, 52.6% exhibited low adherence, followed by 20.4% with moderate adherence and 27.1% with high adherence to antidiabetic medications. Socio-demographic characteristics, including age, gender, marital status, and education, showed no significant association with non-adherence. Several clinical and behavioral factors, however, were significantly related to adherence patterns. Higher odds of non-adherence were observed among patients who did not benefit from therapeutic education (p=0.009), individuals not performing self-monitoring of blood glucose (p<0.001), and those using phytotherapy (p=0.03). Additional determinants of poor adherence included higher out-of-pocket medication costs (p=0.043), drug shortages in health facilities (p=0.013), forgetfulness (p<0.001), absence of diabetic symptoms (p<0.001), and psychological barriers such as mistrust in physicians (p<0.001). Conversely, better adherence was associated with the combined use of oral antidiabetic drugs and insulin, follow-up with a general physician (p=0.036), dietary compliance (p<0.001), and regular physical activity (p=0.021).

**Conclusion:**

medication non-adherence remains highly prevalent among patients with type 2 diabetes. Our findings highlight the importance of strengthening therapeutic education, improving access to medications, and promoting healthy lifestyle behaviors to enhance adherence. Targeted interventions addressing both behavioral and healthcare system-related barriers are essential to improve long-term diabetes outcomes in the Moroccan context.

## Introduction

Type 2 diabetes (T2D) is a major public health problem and is rapidly becoming a global health crisis. Its prevalence continues to increase worldwide [1,2]. During the past three decades, the global burden of diabetes has increased dramatically, making it one of the leading causes of mortality. Currently, approximately one in eleven adults worldwide lives with diabetes, and the vast majority of these cases, around 90%, are T2D [3]. Compared with high-income countries, the rise in diabetes disproportionately affects low and middle-income settings, where it contributes to higher levels of morbidity, mortality, and healthcare costs [4]. According to the International Diabetes Federation (IDF), the Middle East and North Africa (MENA) region counted an estimated 85 million adults living with diabetes in 2024, a figure projected to reach 162.6 million by 2050 [5].

Type 2 diabetes (T2D) is now a major public health problem in Morocco. National estimates suggest that T2D has affected 6.6% of the population aged 20 years and older in 2000 and 12.4% in 2016 [6,7].

The management of this chronic metabolic disease is a major challenge due to its various complications and consequences, mainly due to a lack of compliance with anti-diabetic drugs, which remains a concern [8]. Effective management of T2D depends on controlling glycemia within normal ranges. It is well-known that any deterioration in glucose control leads to a rise in a person's risk of morbidity and mortality. Increased healthcare costs are also created due to these complications, including retinopathy, nephropathy, and neuropathy [9]. The risk of these complications and comorbidities can be significantly reduced when patients adhere consistently to their prescribed anti-diabetic medications and adopt healthier lifestyle practices [10]. Furthermore, anti-diabetic medications help stabilize blood glucose levels, preventing fluctuations between hyperglycemia and hypoglycemia that can adversely affect the overall health and well-being of a patient [11].

Medication non-adherence among patients with T2D poses a significant barrier to effective disease management and is closely linked to poorer clinical outcomes. Several studies have been conducted to evaluate drug adherence among diabetic patients, revealing critical factors that contribute to non-adherence and identifying potential intervention targets [12]. A systematic review of 16 studies in North Africa focused on adherence rates to treatment found a combined nonadherence rate of 38%, with significant heterogeneity among the studies. Factors associated with non-adherence in type 2 diabetes included education level, social coverage, therapeutic education, cost of medication, socioeconomic status, duration of diabetes, unbalanced diabetic diets, polypharmacy, female gender, family support, and age [13]. A recent study in Morocco found that 23.3% of patients with T2D do not adhere to medication. The study identified several key factors influencing adherence, including access to treatment, side effects, family support, disease duration, age, and patients´ awareness about their condition [14]. These findings underscore the need for a comprehensive understanding of the factors associated with medication non-adherence among patients with T2D, particularly within Moroccan healthcare settings such as the University Hospital Center of Casablanca, to develop targeted interventions and improve patient outcomes. Therefore, this current study aims to assess the prevalence of medication non-adherence and provide a detailed analysis of the factors linked to non-adherence among patients with T2D at the University Hospital Center of Casablanca. By drawing on existing research and empirical evidence, this study seeks to inform effective strategies for improving medication adherence and optimizing the management of T2D in this context.

To achieve these objectives, the following research questions were formulated: 1) What is the prevalence of medication non-adherence among Moroccan patients with T2D, based on data from a tertiary hospital in Casablanca? 2) Which socio-demographic, clinical, and treatment-related factors are associated with medication non-adherence in this population? 3) How do behavioral and healthcare system factors influence medication adherence among these patients?

Based on previous findings and theoretical frameworks, two research hypotheses were proposed: H1: medication non-adherence among patients with T2D is significantly associated with socio-demographic, clinical, and treatment-related factors; H2: behavioural factors and limitations of the healthcare system reduce medication adherence among Moroccan patients with T2D.

## Methods

**Study setting:** the study was carried out at the Department of Endocrinology, Diabetology, and Metabolic Diseases at Ibn Rochd University Hospital in Casablanca, Morocco. This university hospital, with a total capacity of 1,685 beds, comprises three major hospitals, the largest being Ibn Rochd Hospital with 1,020 beds [15]. As a tertiary hospital, it offers comprehensive medical and surgical care, including specialised emergency and elective services. The Department of Endocrinology in Casablanca is the second largest endocrinology unit in Morocco [16]. It provides care for patients with diabetes and a broad spectrum of endocrine and metabolic disorders. For this study, patients with type 2 diabetes who were receiving inpatient care, outpatient consultations, or day-hospital management at the Endocrinology Department of Ibn Rochd Hospital were consecutively recruited during their regular follow-up visits.

**Figure 1:**
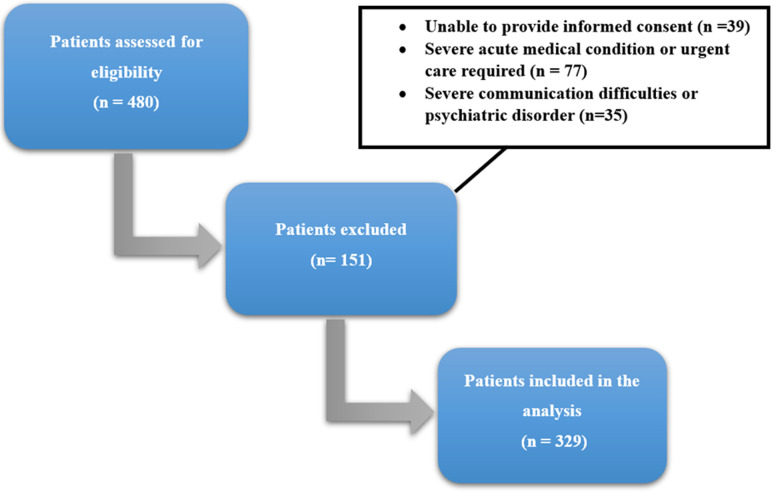
flow diagram of patient recruitment and inclusion in the study

**Study design and population:** this quantitative cross-sectional study was conducted from May to October 2023, involving 329 patients with type 2 diabetes in the Endocrinology Department of the Ibn Rochd University Hospital in Casablanca, Morocco. The inclusion criteria consisted of patients over 18 years old, having a diagnosis of type 2 diabetes for six months or more, and under oral antidiabetic medication and/or insulin for at least 3 months. The researchers obtained informed consent from all participants. The exclusion criteria were as follows: patients with type 1 or gestational diabetes, those with severe acute medical conditions, or those requiring urgent medical care were excluded from the study. Participants who were unable to provide informed consent were also excluded. Similarly, individuals with severe communication difficulties (such as significant speech or hearing impairment) or a documented history of psychiatric disorders were not included (Figure 1).

**Sample size determination:** the sample size was calculated using the single-proportion formula [17]:

$$n=\frac{Z^{2}xPx\left(1-P\right)}{d^{2}}$$

Where n is the required sample size, Z is the standard normal deviate corresponding to a 95% confidence level (Z=1.96), p is the estimated prevalence of diabetes, and d is the margin of error set at 5%.

The value of p was derived from the World Health Organization estimate, indicating a diabetes prevalence of 12.4% in Morocco [7]. Based on these parameters, the calculation yielded a minimum required sample size of 167 participants.

$$n=\frac{1.96x0.124x\left(1-0.124\right)=167}{0.05^{2}}$$

**Sampling procedure:** a convenience sampling technique was used for participant recruitment. Eligible patients were identified from the daily consultation and hospitalization lists at the University Hospital Center of Casablanca. Patients who met the inclusion criteria were invited to participate in the study and informed of the study objectives during their routine appointments. Participants who voluntarily agreed to participate provided written informed consent before enrolment. Data collection was conducted by trained healthcare professionals with backgrounds in nursing and public health under the supervision of the principal investigators. Each interview was conducted in a private setting and lasted approximately 30 to 45 minutes.

**Sociodemographic and clinical data:** data were collected from two complementary sources: patients´ medical records and a structured questionnaire administered by an interviewer. Before data collection, the questionnaire was pre-tested in a pilot sample of 20 patients with type 2 diabetes attending the same hospital to evaluate the clarity, relevance, and comprehensibility of the items. Minor linguistic and formatting adjustments were made based on participants´ feedback to ensure content validity and ease of understanding. Data from the pilot phase were not included in the final analysis. Sociodemographic variables included age, sex, marital status, educational level, employment status, place of residence (urban/rural), and health coverage. Lifestyle-related factors such as smoking habits, physical activity, dietary compliance, and the use of phytotherapy for diabetes management were also recorded.

Clinical and biological data, such as duration of diabetes, glycated haemoglobin level (HbA1c), type of antidiabetic therapy, and presence of comorbidities or complications, were extracted from medical files using a standardised data extraction form. Blood pressure was measured in a seated position after at least five minutes of rest using a calibrated digital sphygmomanometer, and the mean of two consecutive readings was recorded. Body weight and height were measured with participants wearing light clothing and no shoes. Body mass index (BMI) was calculated as weight in kilograms divided by height in meters squared (kg/m^2^) and classified according to World Health Organization criteria as underweight (<18.5), normal (18.5-24.9), overweight (25.0-29.9), or obese (≥30.0) [18].

**Assessment of self-reported reasons for non-adherence:** participants were asked to indicate the main difficulties encountered in taking their antidiabetic medications using a structured question comprising several predefined items (e.g., high cost of drugs, unavailability at health centers, forgetting doses, injection pain, swallowing problems, disappearance of diabetic symptoms, lack of immediate benefit, and mistrust of physicians). Each item was rated dichotomously (“yes” = presence of the problem, “no” = absence of the problem).

**Assessment of medication adherence:** adherence to antidiabetic treatment was assessed using the eight-item Morisky Medication Adherence Scale (MMAS-8). This tool consists of seven dichotomous (yes/no) questions and one item rated on a five-point Likert scale, yielding a total score of 0-8. Adherence was categorized as high (score of 8), medium (6 to <8), and low (<6). For analysis, patients scoring 6 or above were considered adherent, while those with scores below 6 were classified as non-adherent. The Arabic version of the MMAS-8 has demonstrated satisfactory reliability and validity among patients with type 2 diabetes [19], and the scale has been widely validated across diverse populations and compared with other adherence measures [20]. The use of the 8-item Morisky Medication Adherence Scale (MMAS-8; ©MMAS 2006, Donald E. Morisky, MMAR LLC, USA) in this study was authorised through an official license obtained from Dr. Donald E. Morisky. Permission to use the validated Arabic version was also obtained.

**Data analysis:** data were initially entered into Microsoft Excel and subsequently imported into the Statistical Package for the Social Sciences (SPSS, Version 25) for analysis. The statistical parameters used for descriptive analysis included frequencies, percentages, means, and standard deviations to characterize the study population and report the prevalence of medication non-adherence. The Chi-square (X^2^) test was applied to assess associations between categorical variables, with a p-value<0.05 considered statistically significant. For the assessment of medication adherence, participants with an MMAS-8 score of 6 or higher were classified as adherent, while those with a score below 6 were considered non-adherent. In the bivariate analysis, patients with high adherence were grouped as “adherent,” whereas those with medium or low adherence were categorized as “non-adherent,” to identify factors associated with medication non-adherence. Binary logistic regression was also performed to further explore the impact of study variables on medication adherence. Crude odds ratios (COR) and 95% confidence intervals were calculated to assess the association between each variable and medication adherence. A p-value of 0.05 or less was considered statistically significant.

**Ethical considerations:** this study received ethical authorization from the National Commission for the Control of Personal Data Protection in Morocco. under authorization number A-RS-903/2024. All procedures were conducted in accordance with Moroccan Law 09-08 on the protection of personal data and with the ethical principles of the Declaration of Helsinki. All participants gave their informed consent prior to data collection.

## Results

**Socio-demographic characteristics and lifestyle behaviors of the participants:** a total of 329 patients with type 2 diabetes were included in the study. The majority were women (71.4%), and the mean age was 56.9 years. Most participants were married (65%), unemployed (65.3%), and lived in urban areas (75.7%). Nearly half (47.1%) had no formal education, and 79.9% were covered by health insurance. A family history of diabetes was reported by 72.9% of respondents. Regarding lifestyle behaviors, 66.6% reported no regular physical activity, 45.6% adhered to dietary recommendations, 17% were smokers, and 32.1% reported using phytotherapy (Table 1).

**Table 1:** sociodemographic characteristics and lifestyle behaviors of 329 patients with type 2 diabetes recruited at the Endocrinology Department of Ibn Rochd University Hospital, Casablanca, Morocco (May-October 2023)

Variables	Population (N= 329)	
**Age (years)**	**Frequency**	**Percentage (%)**
18-30	8	2.4
31-50	79	24.0
>50	242	73.6
**Gender**		
Male	94	28.6
Female	235	71.4
**Marital status**		
Single	42	12.8
Married	214	65.0
Divorced/widowed	73	22.2
**Education**		
Not educated	155	47.1
Primary school	101	30.7
High school/university	73	22.2
**Employment status**		
Unemployed	215	65.3
Employed	90	27.7
Retired	23	7.0
**Health insurance**		
Insured	263	79.9
Unsured	66	20.1
**Residence**		
Urban	249	75.7
Rural	80	24.3
**Family history of type 2 diabetes**		
Yes	240	72.9
No	89	27.1
**Smoking**		
Yes	56	17.0
No	273	83.0
**Physical activity**		
Not at all	219	66.6
1 to 4 days per week	68	20.7
⩾5 days per week	42	12.8
**Dietary compliance**		
Yes	150	45.6
No	179	54.4
**Phytotherapy**		
Yes	108	32.8
No	221	67.2

**Anthropometric measurements and clinical records of the patients with T2D:** the mean and standard deviation (SD) of the time of diagnosis and the last hemoglobin A1c(HbA1c) test result were 11.7 years ± 7.7 years and 10.43 ± 2.8, respectively. Most patients had diabetes for more than 10 years (52%), and 88.4% had poor HbA1c levels (>7%). Most had comorbidities (62%), primarily hypertension (48%) and dyslipidemia (41.6%). Among the study participants, 67.8% were treated with insulin, either alone or in combination with oral antidiabetic drugs (OAD), while 63.2% were on some form of OAD therapy. The most frequently prescribed OAD was Metformin, taken by 55.6% of patients, followed by Sulfonylurea in 31.9% of cases. A significant proportion of patients were also on statins (36.5%) and other cardiovascular medications. Nearly all patients (96.4%) experienced diabetes-related complications, with hypoglycemia being the most common, affecting 50.2% of the patients. Other notable complications included severe hypoglycemia (50.2%), diabetic retinopathy (30.1%), diabetic nephropathy (11.2%), coronary insufficiency (12.5%), diabetic foot (11.9%), and diabetic ketosis (13.7%) (Table 2).

**Table 2:** clinical characteristics of 329 patients with type 2 diabetes, recruited from the Endocrinology Department of Ibn Rochd University Hospital, Casablanca, Morocco, between May and October 2023

Variables	Population (N= 329)	
	**Frequency ± SD**	**Percentage (%)**
**Diabetes duration (years), mean± SD**	**11.77 ± 7.77**	
6 months-3 years	58	17.6
4-10 years	100	30.4
More than 10 years	171	52
BMI (kg/m2); mean± SD	27.46 ± 6.75	
Normal (<18.5)	103	31.3
Underweight (18.5-24.9)	19	5.8
Overweight (25.0-29.9)	106	32.2
Obese (≥30)	101	30.7
**Blood pressure (mmHg)**		
SBP, mean± SD	133.36 ± 20.14	
DBP, mean± SD	74.48 ± 9.40	
HbA1c; mean± SD	10.44 ± 2.90	
Good ≤7%	38	11.6
Poor > 7%	291	88.4
**Comorbidities**		
Yes	204	62.0
No	125	38.0
**Type of comorbidities**		
Hypertension	158	48.0
Cardiopathy	63	19.1
Dyslipedimia	137	41.6
Depression	50	15.2
**Antidiabetic treatment**		
OAD	106	32.2
Insulin	121	36.8
OAD+insulin	102	31.0
**Type of OAD taken**		
Metformin	183	55.6
Sulfonylure	105	31.9
Sulfonylurea+metformin	85	25.8
DDP4- inhibitors	3	0.9
DDP4- inhibitors+metformin	11	3.3
**Other medication**		
Statins	120	36.5
Beta blockers	47	14.3
ACEI	42	12.8
ARBs	49	14.9
CCB	53	16.1
A combination of antihypertension therapy	24	7.3
APT	59	17.9
Gabapentin	19	5.8
**Presence of diabetes complications**		
Yes	317	96.4
No	12	3.6
**Types of type 2 diabetes complications**		
Severe hypoglycemia	165	50.2
Diabetic ketosis	45	13.7
Diabetic retinopathy	99	30.1
Diabetic nephropathy	37	11.2
Coronary insufficiency	41	12.5
Diabetic foot	39	11.9

ACEI: angiotensin convertor enzyme inhibitor; APT: antiplatelet; ARBs: angiotensin II receptor blockers; BMI: body mass index; CCB: calcium channel blocker; DBP: diastolic blood pressure; DDP4: dipeptidyl peptidase-4; HbA1c: hemoglobin A1c; N: number of cases; OAD: oral antidiabetic drugs; SBP: systolic blood pressure; T2D: type 2 diabetes

**Prevalence of non-adherence to antidiabetic medication as measured by the Morisky Medication Adherence Scale (MMAS-8):** the prevalence of adherence to anti-diabetic medications reveals that 27.1% of participants are classified as high adherers, while 52.6% are low adherers, and 20.4% exhibit medium adherence.

**Association between different factors and non-adherence among the patients with T2D:** the bivariate logistic analysis showed that none of the socio-demographic characteristics, such as age, gender, marital status, education, or employment status, were significantly associated with non-adherence to anti-diabetic medications among patients with T2D. However, patients referred by general physicians were less likely to be non-adherent compared to those referred by endocrinologists (COR=0.627, p=0.036) (Table 3). The results from the bivariate analysis indicated that factors such as BMI, blood pressure, depression, smoking history, and the presence of comorbidities did not demonstrate a significant relationship with non-adherence. In contrast, patients who complied with dietary recommendations were less likely to be non-adherent (OR=0.35, p<0.001). Likewise, those who reported regular physical activity showed significantly lower odds of non-adherence (OR=0.58, p=0.021) (Table 4). In the bivariate analysis, the use of a combination of oral antidiabetic drugs (OAD) and insulin was significantly associated with antidiabetic medication adherence (OR=0.564, p=0.035). This indicates that patients receiving combined therapy were more likely to be adherent. Additionally, a higher frequency of daily medication intake (≥3 doses) was significant (OR=1.692, p=0.049) (Table 5).

**Table 3:** bivariate logistic analysis of the association between sociodemographic characteristics and medication non-adherence among patients with type 2 diabetes recruited in Casablanca, Morocco (N=329)

Variables	Category	Non-adherent N=173(%)	Adherent N=156(%)	COR	95% CI	P-Value
Age(years)	18-30	3 (37.5)	5 (62.5)	1		0.285
	31-50	47(59.5)	32 (40.5)	0.580	0,136-2.483	0.463
	>50	123 (50.8)	119 (49.2)	1.421	0.849-2.378	0.181
Gender	Male	56 (59.6)	38 (40.4)	1		0.109
	Female	117 (49.8)	118 (50.2)	1.486	0.915-2.414	
Marital status	Single	20 (47.6)	22 (52.4)	1		0.325
	Married	119 (55.6)	95 (44.4)	1.043	0.487-2,231	0.924
	Divorced/widowed	34 (46.6)	39 (53.4)	1.437	0.843-2.449	0.183
Education	Primary school	47 (46.5)	54 (53.5)	1		0,309
	Not educated	84 (54.2)	71 (45.8)	0.642	0.350-1.179	0.153
	High school/university	42 (57.5)	31 (42,5)	0.873	0.498-1.531	0.636
Employment status	Employed	108 (50.2)	107 (49,8)	1		0.45
	Unemployed	52 (57.8)	38 (42.2)	0.776	0.326-1.847	0.567
	Retired	13 (56,5%)	10 (43.5)	1.053	0.418-2.653	0.913
Referring physician	Endocrinologist	82 (47.1%)	92 (52.9)	1		
	General physician	91 (58.7%)	64 (41.3)	0,627	0.405-0.970	0.036*

CI: confidence intervals; COR: crude odds ratio; N: number of cases; %: percentage; *p<0.05

**Table 4:** association between medication non-adherence, anthropometric measures, and lifestyle behaviors among patients with type 2 diabetes at Ibn Rochd University Hospital, Casablanca, Morocco, between May and October 2023

Variables	Category	Non-adherent N=173(%)	Adherent N=156(%)	COR	95% CI	P-Value
BMI	Normal	54 (52.4)	49 (47.6)	1		0.892
	Underweight	11 (57.9)	8 (42.1)	1.010	0.629-1.621	0.966
	Overweight/obese	108 (52.2)	99 (47.8)	1.260	0.487-3.261	0.633
Blood pressure	Normal	92 (48.9)	81 (57.4)	1		0.126
	Hypertension	96 (51.1)	60 (42.6)	0.710	0.457-1.102	
Depression antecedent	Yes	30 (60)	20 (40)	1		0.256
	No	143 (51.3)	136 (48.7)	0.701	0.380-1.293	
Smoking antecedent	Yes	34 (60.7)	22 (39.3)	1		0.183
	No	139 (50.9)	134 (40.1)	1.490	0.829-2.678	
Presence of comorbidities	Yes	107 (52.5)	97 (47.5)	1		0.951
	No	66 (52.8)	59 (47.2)	0.980	0.631-1.540	
Hypertension	Yes	82 (51.9)	76 (48.1)	1		0.811
	No	91 (53.2)	80 (46.8)	1.050	0.684-1.626	
Cardiopathy	Yes	33 (52.4)	30 (47.6)	1		0.971
	No	140 (52.6)	126 (47.4)	0.990	0.571-1.716	
Dyslipidemia	Yes	67 (48.9)	70 (51.1)	1		0.259
	No	106 (55.2)	86 (44.8)	0.777	0.5-1.205	
Dietary compliance	No	115 (64.2)	64 (35.8)	1		0.000*
	Yes	58 (38.7)	92 (61.3)	0.351	0.224-0.550	
Physical activity	No	125 (57.1)	94 (42.9)	1		0.021*
	Yes	48 (43.6)	62 (56.4)	0.582	0.367-0.924	

CI: confidence intervals; COR: crude odds ratio; N: number of cases; %: percentage; *p<0.05

**Table 5:** bivariate logistic analysis of the association between treatment-related characteristics and medication non-adherence among patients with type 2 diabetes in Casablanca, Morocco (N=329)

Variables	Category	Non-adherent N=173(%)	adherent N=156(%)	COR	95% CI	P-Value
Diabetes duration (years)	6 months-3 years	27 (46.6)	31 (53.4)	1		0.440
	4-10 years	57 (57.0)	43 (43.0)	0.802	0.442-1.458	0.470
	More than 10 years	89(52.0)	82 (48.0)	1.221	0.743-2.007	0.430
HbA1c levels	Good ≤7%	21 (55.3)	17 (44.7)	1		0.725
	Poor >7%	152 (52.2)	139 (47.8)	1.130	0.573-2.229	
Antidiabetic treatment	OAD	59 (55.7)	47 (44.3)	1		0.081
	Insulin	54 (44.6)	67 (55.4)	0.879	0.507-1.523	0.645
	OAD + insulin	60 (58.8)	42 (41.2)	0.564	0.331-0.961	0.035*
Total number of drugs taken	1	9 (42.9)	12 (57.1)	1		0.633
	2	49 (56.3)	38 (43.7)	0.614	0.241-1.565	0.307
	3	32 (50.8)	31 (49.2)	1.055	0.605-1.839	0.850
	4	17 (44.7)	21 (55.3)	0.845	0.458-1.556	0.588
	More than 4	66 (55.0)	54 (45.0)	0.662	0.318-1.379	0.271
Frequency of daily medication intake	Single dose	4 (44.4)	5 (55.6)	1		0.134
	2 doses	47 (62.7)	28 (37.3)	0.807	0.212-3.075	0.753
	⩾3 doses	122 (49.8)	123 (50.2)	1.692	0.995-2.877	0.052
Antidiabetic dosage	Fixed dosage	91 (49.7)	92 (50.3)	1		0.245
	Varying dosage	82 (56.2)	64 (43.8)	0.772	0.499-1.195	
Diabetes Complications	Yes	168(53.0)	149(47.0)	1		0.444
	No	5 (41.7)	7 (58.3)	1.579	0.491-5.079	

CI: confidence intervals; COR: crude odds ratio; N: number of cases; %: percentage; *p<0.05

To identify the factors influencing medication adherence, several variables were analyzed. The findings indicate that patients who did not receive therapeutic education were more likely to be non-adherent than those who did (COR=1.79; p=0.009). Likewise, the absence of self-monitoring of blood glucose was strongly associated with non-adherence (COR=2.39; p<0.001). The use of phytotherapy was also significantly linked to higher odds of non-adherence (p=0.03). In contrast, treatment-related side effects showed only a borderline association with non-adherence (p=0.053) (Table 6). Self-reported reasons for non-adherence revealed several significant associations. High medication costs (p=0.043), unavailability of drugs in health centers (p=0.013), and forgetting to take medication (p<0.001) were strongly associated with non-adherence. In addition, the disappearance of diabetic symptoms (p<0.001) and mistrust toward healthcare providers (p<0.001) showed significant links with higher non-adherence. Other reported reasons, such as swallowing difficulties, lack of immediate treatment benefits, and pain on injection, were not statistically associated with non-adherence (Table 7).

**Table 6:** associations between medication non-adherence and therapeutic education, behavioral, and treatment-related factors among 329 patients with type 2 diabetes recruited at Ibn Rochd University Hospital, Casablanca, Morocco (May-October 2023)

Variables	Category	Non-adherent N=173(%)	Adherent N=156(%)	COR	95% CI	P-value
Benefits of therapeutic education	Yes	64 (44.4)	80(55.6)	1		0.009*
	No	109 (58.9)	76 (41.1)	1.793	1.154-2.784	
Side effects related to the treatment	Yes	86 (58.5)	61 (41.5)	1		0.053*
	No	87 (47.8)	95 (52.2)	0.650	0.419-1.007	
Adherence to self-monitoring of blood glucose	Yes	51 (39.5)	78 (60.5)	1		0.000*
	No	122 (61)	78 (39.0)	2.392	1.521-3.763	
Phytotherapy	Yes	66 (61.1)	42 (38.9)	1		0.03*
	No	107 (48.4)	114 (51.6)	0.597	0.374-0.954	

CI: confidence intervals; COR: crude odds ratio; N: number of cases; %: percentage; *p<0.05

**Table 7:** self-reported reasons for medication non-adherence among patients with type 2 diabetes included in the cross-sectional study conducted in Casablanca, Morocco, between May and October 2023 (N=329)

Factors	Frequency (%) N=329	Non-adherent N=173(%)	Adherent N=156(%)	P-value

The drug is expensive to buy on a daily basis	100 (30.4)	61 (61.0)	39 (39.0)	0.043*
Unavailability of drugs at the health centers	93 (28.3)	59 (63.4)	34 (36.6)	0.013*
Forgetting to take medication	80 (24.3)	58 (72.5)	22 (27.5)	0.000*
Pain on injection	81 (24.6)	45 (55.6)	36 (44.4)	0.537
swallowing disorder	20 (6.1)	11 (55.0)	9 (45.0)	0.823
Lack of immediate benefits of treatment	50 (15.2)	30 (60.0)	20 (40.0)	0.254
Disappearance of diabetic symptoms	44 (13.4)	39 (88.6)	5 (11.4)	0.000*
mistrust in physicians	98 (29.8)	71 (72.4)	27 (27.6)	0.000*

Chi-square test; %: percentage; *p<0.05

## Discussion

Adherence to antidiabetic medication plays a crucial role in ensuring adequate glycemic control and preventing both acute and long-term complications. Understanding the determinants of non-adherence is therefore essential to improve therapeutic outcomes, particularly in countries like Morocco, where the burden of T2D continues to rise. In this context, the present study aimed to assess the prevalence of medication non-adherence among Moroccan patients with T2D and identify its associated factors.

Regarding the prevalence of non-adherence, nearly half of the participants presented low adherence to their medication, aligning with the non-adherence rates of 55.3% and 53.5% reported in Egypt and Rwanda, respectively [12,21]. This similarity could be due to comparable study designs. Additionally, our findings indicated that the non-adherence prevalence in our study was higher than that found in prior studies conducted in Saudi Arabia and Kenya [22,23]. Where better adherence was observed. In contrast, some studies, such as those in Ethiopia, have noted even higher non-adherence rates to antidiabetic medications than ours [24]. These variations highlight regional differences in healthcare systems and patient support, underscoring the importance of developing strategies tailored to specific contexts to improve medication adherence.

The issue of adherence to medications in diabetes is multifactorial and extends beyond individual behaviour. As described in the five-dimensional framework of the World Health Organization (WHO), adherence is shaped by disease, treatment, healthcare system, patient, and socio-economic factors [25], several of which were evident in our study.

The present finding did not report a significant association between socio-demographic characteristics and non-adherence to medication among patients with T2D. Similarly, sex did not have a significant influence on medication adherence, consistent with the findings from Sudan and Malaysia [26,27]. However, other studies have shown contrasting results, reporting a higher non-adherence among females in Egypt [28]. In our study, age did not have a significant association with adherence, unlike findings from other settings where adherence increased with age [29,30]. Presumably, these differences may be explained by differences in sample sizes.

Previous studies conducted in Saudi Arabia and Palestine [29,30], reported a positive association between higher education and adherence to medication. However, no significant relationship was found in this study, possibly due to the influence of therapeutic support or family involvement that compensated for lower levels of education. However, Park *et al*. [31] found that in Korea, a higher socioeconomic status was associated with a lower adherence to medication in patients with T2D, which differs from our findings, where socioeconomic factors did not show a significant impact. These differences may be explained by variations in healthcare access, affordability of medications, and patient support systems in countries.

In contrast to Wong *et al*. [32], who reported poorer adherence among patients with comorbidities. This study found no significant association. This may reflect the benefit of more regular medical follow-up and care for patients with comorbidities in our setting. Similarly, while depression was linked to non-adherence in a large Canadian study [33]. It was not a significant factor in our population, possibly due to contextual or methodological differences. The duration of diabetes showed no significant association with medication adherence in this study, unlike findings from Kenya, where an association was reported [34]. Other studies [32,33], however, have shown that patients with a longer duration of diabetes tend to demonstrate better adherence, likely due to greater disease awareness and experience in managing their treatment. Our results also indicate that patients receiving a combination of oral antidiabetic drugs and insulin were less likely to be non-adherent. This supports findings from Rwanda [1,2] but contrasts with those from Cameroon [35], where limited availability and affordability of insulin contribute to poor adherence [35,3,6]. In Morocco, insulin is generally accessible, which may explain the better adherence observed.

Otherwise, better dietary compliance and higher physical activity were significantly associated with improved medication adherence. This is consistent with findings from Tunisia [37]. In contrast, the Malaysian study [38] found that patients with low adherence were more likely to adopt specific dietary restrictions, which were also significantly associated with regular exercise and foot care, reflecting a compensatory rather than supportive strategy. These contrasting findings suggest that lifestyle behaviors can either complement or substitute medication, depending on patients´ perceptions and context. Additionally, the findings of this research revealed that the absence of therapeutic education and poor adherence to self-monitoring of blood glucose were strongly associated with medication non-adherence. This result is consistent with findings from Tunisia and Nigeria [39,40], where patients with better diabetes knowledge and self-management practices were more likely to adhere to their treatment. Evidence also indicates that training in glucometer use improves adherence, underscoring the crucial role of structured educational programs in strengthening patients´ engagement and promoting effective diabetes management. Also, the non-use of phytotherapy in our study was associated with a reduced risk of medication non-adherence among patients with T2D. Similarly, a study in Sudan [41] identified the use of herbal medicine and the unavailability of medications as key barriers to adherence. This research showed that patients referred by general physicians were less likely to be non-adherent, possibly due to easier access and continuity of care in primary health centers. However, another study from Iran reported that higher adherence is associated with more frequent specialist visits [42]. This contrast suggests that accessibility and follow-up regularity may influence adherence differently depending on the health-care setting. Indeed, our results revealed that mistrust toward the physician increased the likelihood of non-adherence. This aligns with previous studies [43,44] showing that limited engagement with physicians or other health professionals is associated with poor medication adherence, whereas higher levels of trust are linked to better adherence. Strengthening the physician-patient relationship through regular follow-up visits and open discussions about self-care difficulties may therefore enhance adherence in patients with T2D.

The results show that high medication costs and drug unavailability are key contributors to non-adherence. Most patients were treated with metformin and sulfonylureas, while newer antidiabetic agents were rarely prescribed, likely due to their higher price. This pattern aligns with findings from Saudi Arabia, where metformin was the most commonly used drug because of its affordability, while newer agents such as liraglutide were significantly more expensive [45]. Similarly, a study from Tanzania reported that high medication costs were strongly associated with non-adherence among patients with T2D [46]. These economic constraints clearly shape prescribing practices and directly impact patients´ ability to adhere to treatment.

This study has several limitations that should be acknowledged. First, it was conducted in a single tertiary hospital in Casablanca, which may limit generalisability, and important factors such as family support, cultural beliefs, and health literacy known to influence adherence were not assessed. Future studies should integrate these dimensions to provide a more comprehensive understanding of adherence. Despite these limitations, the present study has notable strengths. It includes a large and diverse sample of 329 patients from both urban and rural areas, enhancing the relevance of the findings. The use of the validated MMAS-8 scale strengthens the reliability of adherence measurement. Finally, the multidimensional analysis of sociodemographic, clinical, and treatment-related factors provides valuable insight into key determinants of adherence among patients with type 2 diabetes.

## Conclusion

This study indicates that medication adherence among Moroccan patients with T2D remains suboptimal, with a non-adherence prevalence of 52.6%. Medication adherence is influenced by a combination of behavioral, therapeutic, and healthcare system-related factors. Barriers such as multiple daily dosing, high medication costs, irregular drug availability in health centers, mistrust toward healthcare providers, and the use of phytotherapy were strongly associated with non-adherence. In contrast, regular follow-up with a general practitioner, participation in therapeutic education programs, routine self-monitoring of blood glucose, and healthier lifestyle habits, including physical activity and dietary compliance, emerged as important facilitators of adherence. Improving access to affordable antidiabetic treatments and ensuring their continuous availability are essential steps to strengthen adherence. Simplifying treatment regimens and reinforcing patient education should also be prioritized. Moreover, enhancing the patient-provider relationship may help address psychological barriers and promote better engagement with therapy. Integrating digital health solutions, such as mobile applications for reminders, education, and glucose monitoring, may offer a promising complementary approach to support long-term treatment adherence. Together, these strategies could contribute to improved glycemic control and better outcomes for patients with T2D in Morocco.

### What is known about this topic

- Medication adherence in T2D is globally suboptimal and influenced by behavioral and healthcare system factors;
- In low- and middle-income countries, financial constraints and limited access to medications are major barriers to adherence;
- Lifestyle factors and the patient-physician relationship play critical roles in improving medication adherence.

### What this study adds

- This study provides updated evidence on medication adherence levels among Moroccan patients with T2D, revealing low overall adherence (52.6%);
- It identifies key behavioral and system-related determinants of non-adherence, including mistrust toward providers and the use of phytotherapy;
- It highlights protective factors such as therapeutic education, general practitioner follow-up, lifestyle improvements, and self-monitoring of blood glucose; it suggests concrete strategies, such as simplifying treatment regimens, improving medication availability, and integrating digital tools to enhance adherence in the Moroccan context.

## References

[1] El-Kebbi IM, Bidikian NH, Hneiny L, Nasrallah MP (2021). Epidemiology of type 2 diabetes in the Middle East and North Africa: Challenges and call for action. World J Diabetes.

[2] Guariguata L, Whiting DR, Hambleton I, Beagley J, Linnenkamp U, Shaw JE (2014). Global estimates of diabetes prevalence for 2013 and projections for 2035. Diabetes Res Clin Pract.

[3] Ong KL, Stafford LK, McLaughlin SA, Boyko EJ, Vollset SE, Smith AE (2023). Global, regional, and national burden of diabetes from 1990 to 2021, with projections of prevalence to 2050: a systematic analysis for the Global Burden of Disease Study 2021. Lancet.

[4] Moradi-Lakeh M, Forouzanfar MH, El Bcheraoui C, Daoud F, Afshin A, Hanson SW (2017). High Fasting Plasma Glucose, Diabetes, and Its Risk Factors in the Eastern Mediterranean Region, 1990-2013: Findings From the Global Burden of Disease Study 2013. Diabetes Care.

[5] International Diabetes Federation IDF Middle-East and North Africa.

[6] Tazi MA, Abir-Khalil S, Chaouki N, Cherqaoui S, Lahmouz F, Sraïri JE (2003). Prevalence of the main cardiovascular risk factors in Morocco: results of a National Survey, 2000. J Hypertens.

[7] World Health Organization (2016). Global report on diabetes.

[8] Huang J, Ding S, Xiong S, Liu Z (2021). Medication Adherence and Associated Factors in Patients With Type 2 Diabetes: A Structural Equation Model. Front Public Health.

[9] Ohkubo Y, Kishikawa H, Araki E, Miyata T, Isami S, Motoyoshi S (1995). Intensive insulin therapy prevents the progression of diabetic microvascular complications in Japanese patients with non-insulin-dependent diabetes mellitus: a randomized prospective 6-year study. Diabetes Res Clin Pract.

[10] AlQarni K, AlQarni EA, Naqvi AA, AlShayban DM, Ghori SA, Haseeb A (2019). Assessment of Medication Adherence in Saudi Patients With Type II Diabetes Mellitus in Khobar City, Saudi Arabia. Front Pharmacol.

[11] Alqarni AM, Alrahbeni T, Qarni AA, Qarni HMA (2018). Adherence to diabetes medication among diabetic patients in the Bisha governorate of Saudi Arabia-a cross-sectional survey. Patient Prefer Adherence.

[12] Murwanashyaka J de D, Ndagijimana A, Biracyaza E, Sunday FX, Umugwaneza M (2022). Non-adherence to medication and associated factors among type 2 diabetes patients at Clinique Medicale Fraternite, Rwanda: a cross-sectional study. BMC Endocr Disord.

[13] Achouri MY, Tounsi F, Messaoud M, Senoussaoui A, Ben Abdelaziz A (2021). Prevalence of poor medication adherence in type 2 diabetics in North Africa. Systematic review and meta-analysis. Tunis Med.

[14] Houguig K, Rkha S, Rayadi M, Ouzennou N (2022). Factors Influencing Therapeutic Observance in Diabetic Subjects in the Province of Essaouira (Morocco): A Cross-Sectional Study. Ethiop J Health Sci.

[15] Système de santé et offre de soins (2015).

[16] Centre Hospitalo-Universitaire Ibn Rochd Service d’endocrinologie diabetologie maladies metabolique et nutrition.

[17] Pourhoseingholi MA, Vahedi M, Rahimzadeh M (2013). Sample size calculation in medical studies. Gastroenterol Hepatol Bed Bench.

[18] World Health Organization (2025). Obesity and overweight.

[19] Ashur ST, Shamsuddin K, Shah SA, Bosseri S, Morisky DE (2015). Reliability and known-group validity of the Arabic version of the 8-item Morisky Medication Adherence Scale among type 2 diabetes mellitus patients. East Mediterr Health J.

[20] Krousel-Wood M, Islam T, Webber LS, Re RN, Morisky DE, Muntner P (2009). New medication adherence scale versus pharmacy fill rates in seniors with hypertension. Am J Manag Care.

[21] Ali RA, Hamed EN, Al-Torky MA, Atia FM (2021). Medication Adherence and Predictors of Non-Adherence among Patients with Type 2 Diabetes Mellitus in Sohag, Egypt. The Egyptian Journal of Community Medicine.

[22] Almetrek M, Alqahtani M, Al Mudawi A, Assiri A, Alfayi A, Alotaibi N (2023). Adherence of Type 2 Diabetic Patients to Antidiabetic Medications and its Associated Factors in Najran Armed Forces Hospital. Saudi Arabia. MEWFM.

[23] Waari G, Mutai J, Gikunju J (2018). Medication adherence and factors associated with poor adherence among type 2 diabetes mellitus patients on follow-up at Kenyatta National Hospital, Kenya. The Pan African Medical Journal.

[24] Abate TW (2019). Medication non-adherence and associated factors among diabetes patients in Felege Hiwot Referral Hospital, Bahir Dar city administration, Northwest Ethiopia. BMC Res Notes.

[25] World Health Organization, WHO (2003). Adherence to Long-Term Therapies: Evidence for Action, 2003.

[26] Mirghani HO (2019). An evaluation of adherence to anti-diabetic medications among type 2 diabetic patients in a Sudanese outpatient clinic. The Pan African Medical Journal.

[27] Ahmad NS, Ramli A, Islahudin F, Paraidathathu T (2013). Medication adherence in patients with type 2 diabetes mellitus treated at primary health clinics in Malaysia. Patient Prefer Adherence.

[28] Nashat Hegazy N (2017). Quality of Care and Medication Adherence among Patients with Type 2 Diabetes Mellitus. The Egyptian Family Medicine Journal.

[29] Khan AR, Al-Abdul Lateef ZN, Al Aithan MA, Bu-Khamseen MA, Ibrahim IA, Khan SA (2012). Factors contributing to non-compliance among diabetics attending primary health centers in the Al Hasa district of Saudi Arabia. J Family Community Med.

[30] Aker O, Hamooz S, Sweileh W (2004). Rate of compliance among patients with diabetes mellitus and hypertension. An-Najah University Journal for Research-A (Natural Sciences).

[31] Park KA, Kim JG, Kim BW, Kam S, Kim KY, Ha SW (2010). Factors that Affect Medication Adherence in Elderly Patients with Diabetes Mellitus. Korean Diabetes J.

[32] Wong MC, Kong AP, So WY, Jiang JY, Chan JC, Griffiths SM (2011). Adherence to oral hypoglycemic agents in 26,782 Chinese patients: a cohort study. J Clin Pharmacol.

[33] Lunghi C, Zongo A, Moisan J, Grégoire J-P, Guénette L (2017). The impact of incident depression on medication adherence in patients with type 2 diabetes. Diabetes Metab.

[34] Mwangasha FM, Nyamu DG, Tirop LJ (2021). Factors impacting on diabetes knowledge, medication adherence and glycemic control among adult diabetics visiting a county teaching and referral hospital in Kenya: a cross-sectional study. The Pan African Medical Journal.

[35] Aminde LN, Tindong M, Ngwasiri CA, Aminde JA, Njim T, Fondong AA (2019). Adherence to antidiabetic medication and factors associated with non-adherence among patients with type-2 diabetes mellitus in two regional hospitals in Cameroon. BMC Endocr Disord.

[36] Gill G (2014). Diabetes in Africa-Puzzles and challenges. Indian J Endocrinol Metab.

[37] Amara A, Ghammam R, Zammit N, Ben Fredj S, Maatoug J, Ghanam H (2020). Adherence to medication among Tunisian adults with type 2 diabetes. European Journal of Public Health.

[38] Jannoo Z, Mamode Khan N (2019). Medication Adherence and Diabetes Self-Care Activities Among Patients With Type 2 Diabetes Mellitus. Value Health Reg Issues.

[39] Hamdi MS, Elhaj WB, Boukhris I, Nacef IB, Kechaou I, Azzabi S (2021). Observance médicamenteuse chez le diabétique: facteurs déterminants et intérêt de l’éducation thérapeutique du patient. EM-Consulte.

[40] Yusuff KB, Obe O, Joseph BY (2008). Adherence to anti-diabetic drug therapy and self management practices among type-2 diabetics in Nigeria. Pharm World Sci.

[41] Badi S, Abdalla A, Altayeb L, Noma M, Ahmed MH (2020). Adherence to Antidiabetic Medications Among Sudanese Individuals With Type 2 Diabetes Mellitus: A Cross-Sectional Survey. J Patient Exp.

[42] Benrazavy L, Khalooei A (2019). Medication Adherence and its Predictors in Type 2 Diabetic Patients Referring to Urban Primary Health Care Centers in Kerman City. Southeastern Iran. Shiraz E-Medical Journal.

[43] Polonsky WH (2015). Poor medication adherence in diabetes: What´s the problem?. J Diabetes.

[44] Beverly EA, Worley M, Prokopakis K, Ivanov N (2016). Patient-physician communication and diabetes self-care. J Clin Outcomes Manag.

[45] Ali MD, Ahmad A, Banu N, Patel M, Ghosn SA, Eltrafi Z (2023). Evaluation of drug utilisation pattern and cost associated with diabetes mellitusType 2 management in Saudi Arabia. Braz J Pharm Sci.

[46] Rwegerera GM (2014). Adherence to anti-diabetic drugs among patients with Type 2 diabetes mellitus at Muhimbili National Hospital, Dar es Salaam, Tanzania-A cross-sectional study. The Pan African Medical Journal.

